# Optimal Decisions for Two Risk-Averse Competitive Manufacturers under the Cap-and-Trade Policy and Uncertain Demand

**DOI:** 10.3390/ijerph17031010

**Published:** 2020-02-05

**Authors:** Hongxia Sun, Jie Yang, Yang Zhong

**Affiliations:** Business School, Beijing Technology and Business University, Beijing 100048, China; yangjie19950922@163.com (J.Y.); zyang523@163.com (Y.Z.)

**Keywords:** supply chain, cap-and-trade, risk-averse, carbon emission reduction

## Abstract

With the increasingly serious problem of environmental pollution, reducing carbon emissions has become an urgent task for all countries. The cap-and-trade (C&T) policy has gained international recognition and has been adopted by several countries. In this paper, considering the uncertainty of market demand, we discuss the carbon emission reduction and price policies of two risk-averse competitive manufacturers under the C&T policy. The two manufacturers have two competitive behaviors: simultaneous decision making and sequential decision making. Two models were constructed for these behaviors. The optimal decisions, carbon emission reduction rate, and price were obtained from these two models. Furthermore, in this paper the effects of some key parameters on the optimal decision are discussed, and some managerial insights are obtained. The results show that the lower the manufacturers’ risk aversion level is, the higher their carbon emission reduction rate and utilities. As the carbon quota increases, the manufacturers’ optimal carbon reduction rate and utilities increase. Considering consumers’ environmental awareness, it is more beneficial for the government to reduce the carbon quota and motivate manufacturers’ internal enthusiasm for emission reduction. The government can, through macro control of the market, make carbon trading prices increase appropriately and encourage manufacturers to reduce carbon emissions.

## 1. Introduction

Recently, the rise of industrialization has brought great vitality to economic development, but it has also caused a heavy burden on the environment. Global warming poses a great threat to human survival and health, and has aroused widespread concern in the international community [1]. At the current rate of global warming, the average global temperature will rise by more than 4 °C by 2100, making most cities uninhabitable and causing the increased occurrence of natural disasters (https://www.chinadialogue.net/culture/11248-Review-The-Uninhabitable-Earth/en). In 2018, Intergovernmental Panel on Climate Change said that we have a reasonable chance of limiting global warming to 1.5 °C only if we strictly limit greenhouse gas emissions to zero by the 2050s. Industrial production relies heavily on the burning of fossil fuels, the main source of most of the carbon dioxide emissions. The main reason for climate change is greenhouse gases emissions, and most commonly carbon dioxide [2]. Therefore, the question of how to deal with global warming by reducing carbon emissions has become a significant and urgent issue.

To reduce the environmental and climate damage caused by excessive carbon dioxide emissions, governments and international agencies have implemented different regulations to control carbon emissions, of which the cap-and-trade (C&T) policy is the most widely used [3]. The C&T policy refers to the government setting an upper limit for the amount of carbon emission permits available for enterprises. If enterprises need to increase their carbon emissions, then they need to buy permits from enterprises that have excess permits to sell in the carbon trading market [4]. The United States passed the Fourth Amendment to the Clean Air Act in 1990, the Acid Rain Plan. This was the beginning of the C&T system (https://news.mongabay.com/2013/12/making-cap-and-trade-work-the-history-and-future-of-a-proven-program/). The U.S. government has previously focused on reducing emissions in power plants, and because cars, trucks, and aircraft emit more greenhouse gases, nine states in the northeast of the United States and Washington have promised to establish a carbon emissions trading system for the transportation sector to reduce emissions. Since 2008, New Zealand has gradually integrated forestry, liquefied fossil fuels, fixed energy, industry, and agriculture into the New Zealand carbon emission trading system (NZ ETS), and finally achieved the reduction of all sectors and six greenhouse gases in New Zealand by 2015. The EU emissions trading system (EU ETS) was founded in 2005 and is the largest international carbon trading system in the world; its turnover accounts for the vast majority of international carbon trading. There has been a marked decline in carbon emissions covered by the EU ETS, which is also promoting emissions trading in other countries and regions (https://ec.europa.eu/clima/policies/ets_en). In January 2020, the Korean carbon emission trading market was launched in the Pusan, Korea securities and futures exchange as scheduled, making it the second largest carbon trading market in the world. As a major carbon emitter, China’s energy saving and emission reduction are urgent. The C&T regulation has become a very important measure in achieving this goal in China. The carbon trading market was officially launched in 2011 and is scheduled to be rolled out nationwide by 2020 [5]. The C&T scheme is beneficial for environment and enterprises, as setting a carbon cap will slow down climate change, and the carbon trading market will provide additional revenue to companies that can reduce carbon emissions at a lower cost [6], which provides a flexible market mechanism and feasible emission reduction plan for the manufacturing industry [7]. Therefore, this policy has been internationally recognized and widely implemented.

Low carbon production is a production process that aims to reduce greenhouse gas emissions and reduce carbon emission intensity as much as possible through the effective use of clean energy [8], and enterprises need to increase investment in low-carbon technologies to cut down carbon emissions [9]. Some enterprises are actively responding to environmental protection and have already invested in low-carbon technologies. McDonald’s uses recycled biodiesel extracted from cooking oil to power transportation vehicles, saving 7000 tons of carbon dioxide per year, and its promise to reduce greenhouse gas emissions by 36% in 2030 is expected to be kept (https://www.mcdonalds.com/gb/en-gb/newsroom/article/global_commitment.html). KFC has pledged to buy 100% recycled or reusable plastic for its consumer products by 2025 and previously pledged to buy 100% fiber packaging from renewable sources by 2020 (https://www.restaurantdive.com/news/kfc-commits-to-using-more-sustainable-packaging-by-2025/546850/). The competition among enterprises is no longer purely a price competition, because the emergence of low-carbon products, it has been gradually transformed into a low-carbon level and price competition. Therefore, studying the competition among enterprises against the background of carbon emission has not only important theoretical significance, but also profound practical significance [10].

Although low-carbon production has brought much competitive vitality to enterprises, the risks that these enterprises face cannot be ignored. On the one hand, enterprises are faced with the risk of technology research and development failure; even though they have invested a great deal of money, they cannot achieve the ideal effect of emission reduction. On the other hand, they face the risk that the low-carbon products they produce will not be accepted by consumers because consumers may consider several factors when purchasing cleaning products, such as price and availability. Low-carbon products are more expensive than conventional ones because of the high cost of investment in carbon emission reduction technologies, which may reduce the demand for these products; thus, there is much uncertainty about the demand for low-carbon products, leading to greater risks for manufacturers. Moreover, manufacturers’ risk attitude will have some impact on their optimal decision making. Therefore, it is significant to consider the risk attitude of manufacturers under the uncertainty of market demand.

In this paper, considering two risk-averse competitive manufacturers of different sizes, we will analyze the optimal decisions under the C&T policy. This paper mainly attempts to explore the following problems: (1) What are the optimal price and carbon emission reduction rate decisions of the two manufacturers in two different competition models? (2) What are the influences of the risk aversion level, carbon trading price, carbon quota and production cost on manufacturers’ optimal strategies?

The remainder of this paper is presented as follows: Section 2 reviews some of the literature related to our study; Section 3 puts forward the basic hypothesis and establishes the model; in Section 4, the price and carbon emission reduction policies of the two competing manufacturers are studied under the Cournot and Stackelberg behavior models respectively; Section 5 provides a discussion and analysis of the results and uses numerical examples to analyze the influence of the risk aversion level and C&T policy on the optimal strategy; and finally, Section 6 provides conclusions, suggestions and outlooks.

## 2. Literature Review

This paper focuses on a comparison of the optimal solutions for the two risk-averse manufacturers under the C&T policy. Thus, this section reviews and summarizes the literature related to operations management under carbon emissions regulations, risk aversion behavior and competitive behavior in the supply chain (SC).

### 2.1. Operations Management under Carbon Emissions Regulations

As environmental issues have received attention, the literature related to carbon emissions has been widely studied. Many studies have focused on price or production decisions and coordination mechanisms. Jaber et al. [11] investigated the coordination mechanism of one two-level SC including a vendor and a buyer in the context of different carbon emission trading schemes. They discovered a combination of carbon taxes and emissions penalties to be most effective. Gong et al. [12] discussed the production decisions of manufacturers based on the C&T policy, in which manufacturers can choose ordinary technology or green technology for production. He et al. [13] researched the production lot-sizing matters of an enterprise under conditions of different environmental policies. The results showed that optimal emissions are related to the relationship between the cost of setting policies and the cost of maintaining emissions. Xu et al. [14] explored the coproduction and pricing of multi-product manufacturing enterprises under different policies and obtained the optimal cap and tax rate with the goal of the maximization of social welfare. Luo et al. [7] investigated the price strategies of two competing manufacturing firms under the carbon trading mechanism and found that cooperative competition will bring greater profits and less carbon emissions. Dong et al. [15] examined the issue of how to invest in sustainable products under the C&T policy and effectively coordinated this SC adopting revenue sharing contracts. Yang et al. [10] followed the coordination between two competing SCs. By studying horizontal cooperation between chains and vertical cooperation within chains, they found that vertical cooperation can improve the efficiency of emission reduction. Cao et al. [16] studied the production behavior of manufacturers under the policy of a low carbon subsidy, and concluded that the government should give some subsidies in the case of high environmental damage coefficient. Bai et al. [17] studied the problem of excessive carbon emissions in the inventory management of deteriorating products and designed a contract to coordinate the profit in the decentralized system and reduce emissions. Xin et al. [18] used mathematical methods to study the optimal production plans of n countries under the C&T mechanism.

Some studies have considered the influence of various carbon emission policies on the decisions of players. Hua et al. [19] established the economic order quantity (EOQ) model to study the impact of a carbon emission trading system on order quantity, carbon emissions and total cost. Choi [20] explored the optimal purchasing strategies of retailers in the fashion SC under two contracts and found that the carbon tax is of great importance to retailer’s decision-making. Ji et al. [21] developed an optimization model under three carbon emission management mechanisms. By exploring the impact of these schemes on enterprises’ decisions and social welfare, they found that the government could formulate corresponding policies for enterprises with different emissions. Li et al. [4] constructed a Stackelberg game model to investigate the effects of carbon trading policies on manufacturers’ low-carbon production decisions, in which the government is the leader with the goal of maximizing social welfare. Tong et al. [22] established an evolutionary game model to simulate the action choices for each member in the SC and took into account the low-carbon preference of consumers; they also used a Stackelberg model to explore their optimal decisions. Zhang et al. [23] explored the optimal marketing model under the fixed carbon policy and concluded that carbon emissions can be effectively controlled only when the quota and carbon tax rate are within a certain range.

Considering the government’s implementation of carbon emission policies, the above studies mainly focused on the issues of price, competition, coordination and carbon emission reduction of upstream and downstream enterprises, while horizontal competition among enterprises was rarely considered. Although some studies focused on competition between manufacturers, they either did not consider risk aversion or did not consider carbon emissions reduction. In contrast to these works, we will consider the different competition behaviors between risk-averse manufacturers under the C&T policy.

### 2.2. Risk Aversion Behavior in the Supply Chain

Due to market uncertainty and competition, an enterprise’s ability to take risks has an effect on its revenue. Enterprises will consider their ability to take risks when maximizing their expected profits. Many researchers have explored the risk aversion problem in the traditional SC, both in terms of price decision and the impact of risk aversion level. Li et al. [24] applied the conditional value-at-risk (CVaR) criterion to measure the average profits of a manufacturer and a retailer with different risk attitudes. They found that the retailer’s risk aversion level will influence his/her profit share. Liu et al. [25] investigated the optimal price strategy with symmetric information and asymmetric information. They set the risk tolerance level for manufacturers and retailers and discussed the influence of the risk aversion level on price and expected utility. Chen et al. [26] used a principal agent model to study the risk aversion problem in dual channels, and discussed the impact of critical parameters on the retailer’s optimal contract and profit. Choi et al. [27] used game theory to explore the pricing problem where manufacturers and retailers are all risk averse and they explored the impact of demand correlation on profitability. Bai and Meng [28] established the MV framework to explore the operational decision-making of members under two different SC structures and found that low-scale risk aversion is beneficial to players.

Some studies have also taken into account risk aversion in issues related to SC coordination. Gan et al. [29] proposed a flexible contract in three different cases and obtained the optimal sharing rules by characterizing a set of Pareto optimal solutions to effectively coordinate risk-averse agents. Gan et al. [30] found that the previous contracts could not coordinate the SC containing the risk-averse agents, based on which they applied a risk-sharing contract to solve this problem. Wang et al. [31] investigated how gain/loss-sharing-and-buyback (GLB) contracts coordinate a decentralized SC contain a loss-averse retailer in the case of uncertain demand and studied the role of GLB sharing clauses in reducing retailer quantity. Wei and Choi [32] regarded a two-echelon SC including a manufacturer and a risk-averse retailer selling newsstand style fashion products with uncertain market demand. They found that the traditional wholesale price contract was ineffective for coordination. Chiu et al. [33] developed the mean-variance (MV) model to explore the SC under a target sales rebate (TSR) contract. Under the condition of retailer risk avoidance, the supplier solved the SC coordination problem through a two-parameter TSR contract. Zhuo et al. [34] used the MV model to find that option contracts are unable to coordinate the SC while considering risk attitude and analyzed the equilibrium solution of the risk aversion threshold as public or private information. Li and Jiang [35] considered coordination and market competition with risk-neutral suppliers and risk-averse retailers.

The abovementioned papers studied coordination and optimal price strategies considering risk aversion behaviors in the SC, and the risk source is usually inventory management or capital operation. With the emergence of the low-carbon products, risks in the green manufacturing and sales processes have also become an issue to which decision makers must pay attention. Therefore, in contrast to the above works, we will study carbon emissions problems considering risk aversion.

### 2.3. Competitive Behavior in the Supply Chain

With the development of product diversification, the competition among members of the SC on factors such as product price and quality has become increasingly fierce. Some scholars have studied the competitive behavior in the SC. Moorthy [36] studied the competition between two firms over product quality and price, and considered consumers’ preference for high-quality products. Bernstein and Federgruen [37] studied the competition of n retailers in the central system and the decentralized system, and obtained the optimal replenishment strategy of suppliers and the optimal pricing strategy of retailers. Cachon and Kök [38] investigated the competitive problem of multiple manufacturers selling products through one retailer and explored the cooperative behavior of manufacturers and retailers under three different contracts. Lee and Yang [39] established a two-stage game model to study the competition between two suppliers selling alternative products through one retailer and evaluated two different SC contracts. Giri et al. [40] built a Stackelberg game model with retailers as the leader, and discussed the competition issue of product quality among multiple manufacturers under the Cournot and Collusion strategies. The results showed that retailers should develop different pricing strategies for different manufacturers’ products. Huang et al. [41] analyzed the price competition of two-level SC including a manufacturer and duopoly retailers under different power structures, and examined the influence of pricing strategy and power structure on SC performance. Kumar et al. [42] explored the procurement and pricing of two competing retailers under the risk of supply disruption, and provided management suggestions for retailers. They found that retailers pay more attention to reliable supply under their own advantages.

The abovementioned papers studied the price competition and quality competition in traditional SC. But with the emergence of sustainable SC, consumers are paying more attention to the low-carbon level of products. So we consider the competition behavior in the SC under the background of carbon emissions, and study the manufacturers’ carbon emission reduction competition under the C&T policy. In addition, we also consider risk aversion.

Accordingly, reviewing the above three subsections show that many scholars have studied carbon emission reduction, competition or risk aversion problems. However, they considered carbon emissions, competition, and risk aversion separately. In contrast to the abovementioned works, we will explore the price and carbon emission reduction competition between two risk-averse manufacturers under the C&T policy and uncertain market demands. Our research has the following main contributions: (1) The C&T policy and risk aversion are considered in the models under uncertain market demand, and the optimal price and carbon emission reduction strategy were obtained; (2) two different competitive behaviors, simultaneous decision making and sequential decision making, are considered for manufacturers, and (3) numerical analyses of the effect of the parameters on optimal solutions are discussed. Comparisons of the optimal solutions are given for different competitive behaviors.

## 3. Model Descriptions and Assumptions

**Figure 1:**
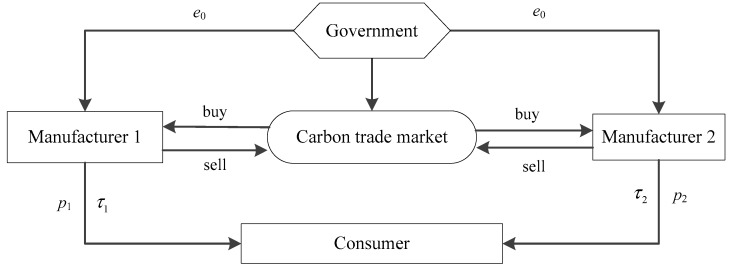
Supply chain structure.

Two competing risk-averse manufacturers (denoted as Manufacturer 1 and Manufacturer 2) in the market produce substitutable products and prepare to invest in low-carbon technology to reduce emissions under the C&T policy. These two manufacturers have two different competitive behaviors: (1) Cournot behavior: simultaneous decision making; and (2) Stackelberg behavior: sequential decision making. The government first allocates carbon emission quotas to manufacturers. Then, these two competitive manufacturers choose their price and carbon emission reduction rate to maximize their utilities. If the manufacturers’ carbon emissions exceed this quota, they ought to buy the excess quota from the carbon trading market, which means paying extra costs. Otherwise, the remaining carbon quota can be sold on the carbon trading market to obtain additional revenue. The SC structure is shown in Figure 1.

The notations used to develop the proposed models are summarized in Table 1.

In order to ensure the feasibility of the model, we make the following assumptions.

**Assumption 1.**  *The Manufacturer 1 is a classic enterprise with relatively large scale in the industry, and Manufacturer 2 is a smaller emerging enterprise. According to the general situation in the market, classic enterprises have been established for a long time and have mastered more customer resources, while emerging enterprises have a weak mass base and low popularity. All of these lead to the market demand of classic enterprises is greater than that of emerging enterprises ($d_{1}>d_{2}$). In addition, since classic enterprises have formed economies of scale, their production cost per unit is lower than that of emerging enterprises ($c_{1}<c_{2}$) [43]. However, because emerging enterprises (Manufacturer 2) usually master advanced technologies, its initial unit carbon emissions are relatively smaller ($e_{1}>e_{2}$)*.

**Assumption 2.**  *Both manufacturers respond to the government’s carbon emission reduction policy and invest in low-carbon technology. The higher the emission reduction rate is, the more difficult the process of reducing emissions, so when the rate increases, the cost of investment will increase sharply. Assume that the input cost function is $I(\tau_{i})=h\tau_{i}^{2}/2$ [44,45,46]. Assume that $\theta >\beta p_{c}e_{i}$, 2ω>θ and $h>\frac{{\left(\theta +\beta p_{c}e_{i}\right)}^{2}}{2\beta }$. In practice, carbon emission reduction technology investment is usually very high, so we assume that h is above a certain level [47]*.

**Assumption** **3.** *The demand for low-carbon products is uncertain. The potential demand for the product is* ${\tilde{d}}_{i}=d_{i}+\varepsilon$ *, where* $d_{i}$ *is the definite part, the continuous variable* ε *is the uncertain part, and* ε~N(0,σ) *[25]. The product demand is linear with the price and emission reduction level of the product itself and its competitors, it can be denoted as*

(1) $${\tilde{q}}_{i}={\tilde{d}}_{i}-\beta p_{i}+\gamma p_{3-i}+\theta \tau_{i}-\omega \tau_{3-i},i=1,2$$

*where* β>γ>0 *and* θ>ω>0 *. The expected quantity* $q_{i}$ *is*

(2) $$q_{i}=E\left({\tilde{q}}_{i}\right)=d_{i}-\beta p_{i}+\gamma p_{3-i}+\theta \tau_{i}-\omega \tau_{3-i},i=1,2$$

**Assumption** **4.** *Manufacturers have different risk aversion levels because of various factors; the larger* $\lambda_{i}$ *is, the more conservative the manufacturer will be. The utility of risk-averse manufacturers can be evaluated by the MV method [35,48,49,50], that is,* $U_{i}=E({\tilde{\pi }}_{i})-\lambda_{i}\sqrt{Var({\tilde{\pi }}_{i})}$ *, where* ${\tilde{\pi }}_{i}$ *is manufacturer i’s uncertain profit,* $E\left({\tilde{\pi }}_{i}\right)$ *is manufacturer i’s expected profit, and* $Var({\tilde{\pi }}_{i})$ *is manufacturer i’s profit variance.*

Based on the above assumptions, manufacturers’ profit function is

(3) $${\tilde{\pi }}_{i}=\left(p_{i}-c_{i}\right){\tilde{q}}_{i}-\frac{1}{2}h\tau_{i}^{2}+\left[e_{0}-\left(1-\tau_{i}\right)e_{i}\right]{\tilde{q}}_{i}p_{c},i=1,2$$

The first item is the profit from product sales, the second item is the cost of low carbon technology investment, and the third item is the income or expenses involved in carbon trading. The expected profit of manufacturer *i* is

(4) $$E({\tilde{\pi }}_{i})=\left[(p_{i}-c_{i})+(e_{0}-e_{i}+\tau_{i}e_{i})p_{c}\right]\left[d_{i}-\beta p_{i}+\gamma p_{3-i}+\theta \tau_{i}-\omega \tau_{3-i}\right]-\frac{1}{2}h\tau_{i}^{2},i=1,2$$

Then, the variance profit of manufacturer *i* is

(5) $$\sqrt{Var({\tilde{\pi }}_{i})}=\sqrt{E{\left[{\tilde{\pi }}_{i}-E({\tilde{\pi }}_{i})\right]}^{2}}=\sigma \left[(p_{i}-c_{i})+(e_{0}-e_{i}+\tau_{i}e_{i})p_{c}\right],i=1,2$$

Hence, the utility of manufacturer *i* is

(6) $$U_{i}=\left[(p_{i}-c_{i})+(e_{0}-e_{i}+\tau_{i}e_{i})p_{c}\right]\left[d_{i}-\beta p_{i}+\gamma p_{3-i}+\theta \tau_{i}-\omega \tau_{3-i}-\lambda_{i}\sigma \right]-h\tau_{i}^{2}/2,i=1,2$$

## 4. The Two Competition Models

### 4.1. Cournot Behavior Model

In this section, manufacturers independently respond with their price and carbon emission reduction rate, their actions are not influenced by others’ decisions, and their optimal price and carbon emission reduction rate can be solved by the simultaneous equation models constructed by their respective response functions.

**Proposition** **1.** $U_{i}$*is a concave function with respect to*$p_{i}$*and*$\tau_{i}$*(*i=1,2*)*.

**Proof.**  Taking the second-order partial derivatives of $U_{i}$ with respect to $p_{i}$ and $\tau_{i}$ (i=1,2), then

$$\frac{\partial^{2}U_{i}}{\partial p_{i}^{2}}=-2\beta ,\frac{\partial^{2}U_{i}}{\partial p_{i}\partial \tau_{i}^{}}=\theta -\beta p_{c}e_{i},\frac{\partial^{2}U_{i}}{\partial \tau_{i}\partial p_{i}^{}}=\theta -\beta p_{c}e_{i},\frac{\partial^{2}U_{i}}{\partial \tau_{i}^{2}}=2p_{c}e_{i}\theta -h.$$

Let $\Delta_{1}$ and $\Delta_{2}$ denote the first and second-order principal minors of Hessian matrix of $U_{i}$, then

$$\Delta_{1}=-2\beta <0,\Delta_{2}=2\beta h-{\left(\theta +\beta p_{c}e_{i}\right)}^{2}>0,$$

By Assumption 2, the second inequality hold, hence the Hessian matrix of $U_{i}$ is negative definite, which implies that $U_{i}$ is a concave function with respect to $p_{i}$ and $\tau_{i}$. □

**Proposition2.**  *In the Cournot behavior model, manufacturer 1 and manufacturer 2’s optimal price and carbon emission reduction rate are*

(7) $$p_{1}^{c}=\frac{2\beta F_{1}+\gamma F_{2}}{\gamma^{2}-4\beta^{2}}-\frac{T_{1}(K_{2}N_{1}-L_{1}N_{2})+S_{2}(K_{1}N_{2}-L_{2}N_{1})}{\left(\gamma^{2}-4\beta^{2})(K_{1}K_{2}-L_{1}L_{2}\right)}$$

(8) $$p_{2}^{c}=\frac{2\beta F_{2}+\gamma F_{1}}{\gamma^{2}-4\beta^{2}}-\frac{T_{2}(K_{1}N_{2}-L_{2}N_{1})+S_{1}(K_{2}N_{1}-L_{1}N_{2})}{\left(\gamma^{2}-4\beta^{2})(K_{1}K_{2}-L_{1}L_{2}\right)}$$

(9) $$\tau_{1}^{c}=\frac{K_{2}N_{1}-L_{1}N_{2}}{K_{1}K_{2}-L_{1}L_{2}}$$

(10) $$\tau_{2}^{c}=\frac{K_{1}N_{2}-L_{2}N_{1}}{K_{1}K_{2}-L_{1}L_{2}}$$

*where $A_{i}=-c_{i}+p_{c}(e_{0}-e_{1})$,*$B_{i}=\theta -\beta p_{c}e_{i}$*,*$E_{i}=2p_{c}e_{i}\theta -h$*,*$F_{i}=-d_{i}+\lambda_{i}\sigma +\beta A_{i}$*,*$G_{i}=-\theta A_{i}$$+p_{c}e_{i}(\lambda_{i}\sigma -d_{i})$*,*$T_{i}=2\beta \omega -B_{i}\gamma$*,*$S_{i}=-2\beta B_{i}+\omega \gamma$*,*$K_{i}=B_{i}S_{i}+T_{i}e_{i}p_{c}\gamma +(\gamma^{2}-4\beta^{2})E_{i}$*,*$L_{i}=$$T_{3-i}(B_{i}+2\beta e_{i}p_{c})$*,*$N_{i}=(\gamma^{2}-4\beta^{2})G_{i}-B_{i}(F_{3-i}\gamma +2\beta F_{i})-e_{i}p_{c}\gamma (F_{i}\gamma +2\beta F_{3-i})$.

**Proof.**  From Proposition 1, the optimal price and carbon emission reduction rate of manufacturer *i* are obtained by solving $\frac{\partial U_{i}}{\partial p_{i}}=0$ and $\frac{\partial U_{i}}{\partial \tau_{i}}=0$ (i=1,2). □

Substituting Equations (7)–(10) into Equations (2) and (6), we can obtain the two manufacturers’ optimal expected quantity and utility.

### 4.2. Stackelberg Behavior Model

In this model, Manufacturer 1 is assumed to be the leader and Manufacturer 2 to be the follower. First, Manufacturer 1 determines the optimal price and the optimal emission reduction rate. Second, Manufacturer 2 decides the optimal price and carbon emission reduction rate on account of the decisions of Manufacturer 1. The way to determine the optimal solution is by using the backward induction method.

**Proposition 3.**  $U_{2}$*is a concave function with respect to*$p_{2}$*and*$\tau_{2}$.

**Proof.**  Taking the second-order partial derivatives of $U_{2}$ with respect to and $\tau_{2}$, then

$$\frac{\partial^{2}U_{2}}{\partial p_{2}^{2}}=-2\beta ,\frac{\partial^{2}U_{2}}{\partial p_{2}\partial \tau_{2}}=\theta -\beta p_{c}e_{2},\frac{\partial^{2}U_{2}}{\partial \tau_{2}\partial p_{2}}=\theta -\beta p_{c}e_{2},\frac{\partial^{2}U_{2}}{\partial \tau_{2}^{2}}=2\theta p_{c}e_{2}-h,$$

Let $\Delta_{1}^{2}$ and $\Delta_{2}^{2}$ denote the first and second-order principal minors of Hessian matrix of $U_{2}$, then

$$\Delta_{1}^{2}=-2\beta <0,\Delta_{2}^{2}=2\beta h-{\left(\theta +\beta p_{c}e_{2}\right)}^{2}>0,$$

By Assumption 2, the second inequality hold, hence the Hessian matrix of $U_{2}$ is negative definite, which implies that $U_{2}$ is a concave function with respect to $p_{2}$ and $\tau_{2}$. □

For any given $\left(p_{1},\tau_{1}\right)$, by solving $\frac{\partial U_{2}}{\partial p_{2}}=0$ and $\frac{\partial U_{2}}{\partial \tau_{2}}=0$, Manufacturer 2’s reaction functions are as follows:

(11) $$p_{2}=\frac{B_{2}G_{2}-E_{2}F_{2}+(p_{c}e_{2}B_{2}-E_{2})(\omega \tau_{1}-\gamma p_{1})}{2\beta E_{2}+B_{2}^{2}}$$

(12) $$\tau_{2}=\frac{B_{2}F_{2}-\beta_{2}G_{2}+(2\beta p_{c}e_{2}+B_{2})(\omega \tau_{1}-\gamma p_{1})}{2\beta E_{2}+B_{2}^{2}}$$

Substituting Equations (11) and (12) into Equation (6), we obtain the utility of Manufacturer 1 as follows:

$$U_{1}=\left(p_{1}+p_{c}e_{1}\tau_{1}+A\right)\left[d_{1}+\left(Q\gamma -\beta \right)p_{1}+\left(\theta -\omega Q\right)\tau_{1}+R-\lambda_{1}\sigma \right]-h\tau_{1}^{2}$$

where $Q=\frac{E_{2}\gamma -p_{c}e_{2}\gamma B_{2}+2\beta p_{c}e_{2}\omega +B_{2}\omega }{2\beta E_{2}+B_{2}^{2}}$ and $R=\frac{B_{2}G_{2}\gamma -E_{2}F_{2}\gamma -B_{2}F_{2}\omega -2\beta G_{2}\omega }{2\beta E_{2}+B_{2}^{2}}$.

**Proposition 4.**  *If*$2(Q\gamma -\beta )J>H^{2}$*, then*$U_{1}$*is a concave function with respect to*$p_{1}$*and*$\tau_{1}$.

**Proof.**  Taking the second-order partial derivatives of $U_{1}$ with respect to $p_{1}$ and $\tau_{1}$, then

$$\frac{\partial^{2}U_{1}}{\partial p_{1}^{2}}=2\left(Q\gamma -\beta \right),\frac{\partial^{2}U_{1}}{\partial p_{1}\partial \tau_{1}}=B_{1}+e_{1}p_{c}\gamma Q-\omega Q,$$

$$\frac{\partial^{2}U_{1}}{\partial \tau_{1}\partial p_{1}}=B_{1}+e_{1}p_{c}\gamma Q-\omega Q,\frac{\partial^{2}U_{1}}{\partial \tau_{1}^{2}}=2e_{1}p_{c}\left(\theta -\omega Q\right)-h$$.

Let $\Delta_{1}^{1}$ and $\Delta_{2}^{1}$ denote the first and second-order principal minors of Hessian matrix of $U_{1}$, respectively, then

$$\Delta_{1}^{1}=2(Q\gamma -\beta )<\frac{\beta^{2}p_{c}e_{2}\gamma \left(p_{c}e_{2}\gamma +2\omega \right)+\theta \gamma \left(2\beta \omega -\theta \gamma \right)}{2\beta \left[{\left(\theta +\beta p_{c}e_{2}\right)}^{2}-2\beta h\right]}<0\;\Delta_{2}^{1}=2\left(Q\gamma -\beta \right)J-H^{2}>0$$

By Assumption 2, the first inequality hold because 2ω>θ and $h>\frac{{\left(\theta +\beta p_{c}e_{i}\right)}^{2}}{2\beta }$, hence the Hessian matrix of $U_{1}$ is negative definite, which implies that $U_{1}$ is a concave function with respect to $p_{1}$ and $\tau_{1}$. □

**Proposition 5.**  *In the Stackelberg behavior model, Manufacturer i’s optimal price and optimal carbon emission reduction rate are*

(13) $$p_{1}^{s}=\frac{\left(e_{1}p_{c}R-G_{1}-\omega QA)H-J(\gamma QA-F_{1}+R\right)}{2J(Q\gamma -\beta )-H^{2}}$$

(14) $$\tau_{1}^{s}=\frac{\left(\gamma QA-F_{1}+R)H-2(Q\gamma -\beta )(e_{1}p_{c}R-G_{1}-\omega QA\right)}{2J(Q\gamma -\beta )-H^{2}}$$

*where*$H=B_{1}-\omega Q+p_{c}e_{1}\gamma Q$*and*$J=E_{1}-2p_{c}e_{1}\omega Q$

(15) $$p_{2}^{s}=\frac{B_{2}G_{2}-E_{2}F_{2}}{2\beta E_{2}+B_{2}^{2}}+\frac{p_{c}e_{2}B_{2}-E_{2}}{2\beta E_{2}+B_{2}^{2}}W$$

(16) $$\tau_{2}^{s}=\frac{B_{2}F_{2}+2\beta G_{2}}{2\beta E_{2}+B_{2}^{2}}+\frac{B_{2}+2\beta p_{c}e_{2}}{2\beta E_{2}+B_{2}^{2}}W$$

*where*$W=\frac{\left(\omega H-\gamma J)(\gamma QA-F_{1}+R)+\left(-H+2\beta \omega -2\gamma \omega \right)(e_{1}p_{c}R-G_{1}-\omega QA\right)}{2J(Q\gamma -\beta )-H^{2}}$.

**Proof.**  From Proposition 3 and Proposition 4, Manufacturer 1’s optimal price and carbon emission reduction rate are obtained by solving $\frac{\partial U_{1}}{\partial p_{1}}=0$ and $\frac{\partial U_{1}}{\partial \tau_{1}}=0$. Manufacturer 2’s optimal decisions can be determined by substituting Manufacturer 1’s optimal solution into his/her reaction function. □

Substituting Equations (13)–(16) into Equations (2) and (6), we can get the two manufacturers’ expected quantity and optimal utility.

## 5. Comparison of the Optimal Solutions and Parametric Sensitivity Analysis

Because of the complexity of these models, we use numerical experiments to examine the effects of carbon trading price $p_{c}$, risk aversion level $\lambda_{i}$, carbon quota $e_{0}$ and production cost $c_{i}$ on optimal solutions for the two different behaviors. In the light of the model description and assumptions in Section 3, the parameter values are given in Table 2.

### 5.1. Effect of Risk Aversion Level on Optimal Solutions

**Figure 2:**
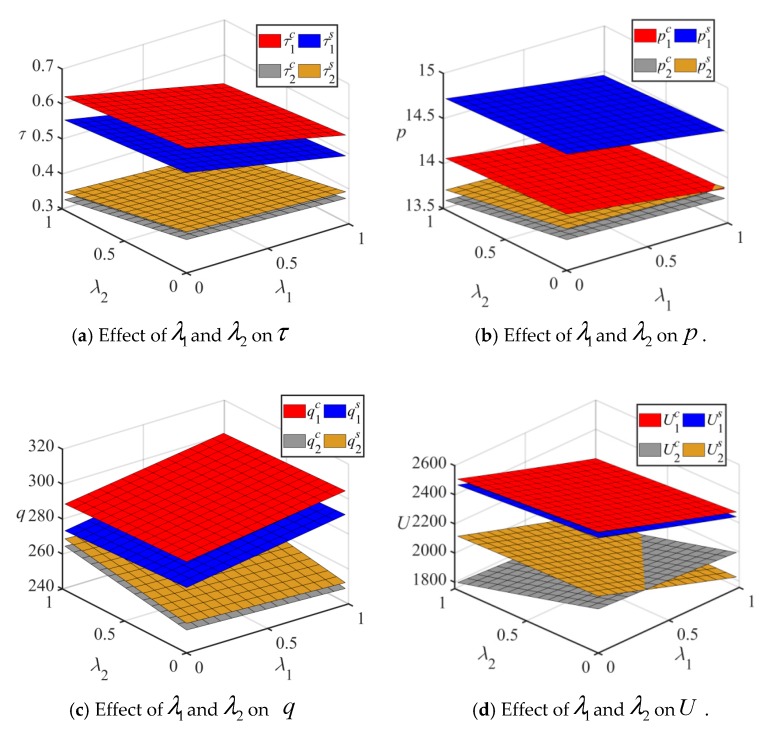
Effect of $\lambda_{i}$ on $\tau_{i}$, $p_{i}$, $q_{i}$, and $U_{i}$.

Based on Table 2, $\lambda_{i}(i=1,2)$ is set between 0 and 1, and the effects of $\lambda_{i}$ on the emission reduction rate, optimal price, expected quantity, and utility for the two different competitive behaviors are shown in Figure 2. Several insights are obtained from Figure 2.

**Insight 1.**  *Regardless of the two competitive models, manufacturers’ emission reduction rate, production price, and utilities decrease in the risk aversion level. However, their expected quantities increase as their own risk aversion level increases and decrease as their rivals’ risk aversion level increases. Therefore, when manufacturers are more optimistic about risk, they prefer to produce low-carbon and environmentally friendly products. Decision makers may lower prices to avoid risk, which may result in the phenomenon that manufacturers reduce prices to attract more consumers. Lower risk aversion levels may increase the utility of manufacturers. Therefore, manufacturers should be optimistic in the face of risks*.

**Insight 2.**  *When manufacturers’ risk aversion levels change, the emission reduction rate relations* $\tau_{1}^{c}>\tau_{1}^{s},\tau_{2}^{s}>\tau_{2}^{c}$ *hold. The optimal price relations* $p_{1}^{s}>p_{1}^{c},p_{2}^{s}>p_{2}^{c}$ *and the expected quantity relations* $q_{1}^{c}>q_{1}^{s}$ *and* $q_{2}^{s}>q_{2}^{c}$ *are also established. For Manufacturer 1, the utility from Cournot behavior is always higher than that from Stackelberg behavior, that is,* $U_{1}^{c}>U_{1}^{s}$ *. However, for Manufacturer 2, when Manufacturer 1 has a lower risk aversion level and Manufacturer 2 has a higher risk aversion level, the utility from the Stackelberg behavior is higher than that of the Cournot behavior, that is,* $U_{2}^{s}>U_{2}^{c}$ *. Therefore, Manufacturer 2 is more willing to be a follower when he/she is conservative, and Manufacturer 1 prefers Cournot behavior over of Stackelberg behavior.*

**Proposition 6.**  *In the Cournot behavior model, when* $c_{1}=c_{2}=c$ *,* $d_{1}=d_{2}=d$ *,* $e_{1}=e_{2}=e$ *and* $\lambda_{1}\neq \lambda_{2}$ *, we can obtain the following:*

*(1) When*$h>\frac{\left(\theta +\beta p_{c}e\right)\left(\theta +\beta p_{c}e+\omega +\gamma p_{c}e\right)}{\gamma +2\beta }$*, if*$\lambda_{1}>\lambda_{2}$*, then*$p_{1}^{c}>p_{2}^{c}$*and*$\tau_{1}^{c}<\tau_{2}^{c}$*; if*$\lambda_{2}>\lambda_{1}$*, then*$p_{2}^{c}>p_{1}^{c}$*and*$\tau_{2}^{c}<\tau_{1}^{c}$.

*(2) When*$h<\frac{\left(\theta +\beta p_{c}e\right)\left(\theta +\beta p_{c}e+\omega +\gamma p_{c}e\right)}{\gamma +2\beta }$*, if*$\lambda_{1}>\lambda_{2}$*, then*$p_{1}^{c}<p_{2}^{c}$*and*$\tau_{1}^{c}>\tau_{2}^{c}$*; if*$\lambda_{2}>\lambda_{1}$*, then*$p_{1}^{c}>p_{2}^{c}$*and*$\tau_{2}^{c}>\tau_{1}^{c}$.

**Proof.**  In Cournot behavior model, when $c_{1}=c_{2}=c$, $d_{1}=d_{2}=d$, $e_{1}=e_{2}=e$ and $\lambda_{1}\neq \lambda_{2}$, from Equations (7)–(10), we get

$$p_{1}^{c}=\frac{\left(-d+\lambda_{1}\sigma +\beta A\right)X-\left(B-w\right)\left[\left(\gamma -2\beta \right)\left(-\theta A+p_{c}e(\lambda_{1}\sigma -d)\right)-\left(B+ep_{c}\gamma \right)\left(-d+\lambda_{1}\sigma +\beta A\right)\right]}{\left(\gamma -2\beta \right)X}\;p_{2}^{c}=\frac{\left(-d+\lambda_{2}\sigma +\beta A\right)X-\left(B-w\right)\left[\left(\gamma -2\beta \right)\left(-\theta A+p_{c}e(\lambda_{2}\sigma -d)\right)-\left(B+ep_{c}\gamma \right)\left(-d+\lambda_{2}\sigma +\beta A\right)\right]}{\left(\gamma -2\beta \right)X}.\;\tau_{1}^{c}=\frac{\left(\gamma -2\beta \right)\left(-\theta A+p_{c}e(\lambda_{1}\sigma -d)\right)-\left(B+ep_{c}\gamma \right)\left(-d+\lambda_{1}\sigma +\beta A\right)}{X}$$

$$\tau_{2}^{c}=\frac{\left(\gamma -2\beta \right)\left(-\theta A+p_{c}e(\lambda_{2}\sigma -d)\right)-\left(B+ep_{c}\gamma \right)\left(-d+\lambda_{2}\sigma +\beta A\right)}{X}$$

where $X=\left(\gamma -2\beta \right)\left(ep_{c}\theta -h\right)+\beta ep_{c}\left[\omega +ep_{c}\left(\gamma -\beta \right)\right]+\theta \left(\omega -\theta \right)$

$$p_{1}^{c}-p_{2}^{c}=\frac{\left(\lambda_{2}-\lambda_{1}\right)\sigma \left(\theta +\beta p_{c}e\right)\left(2\beta p_{c}e+\gamma p_{c}e\right)}{\left(\gamma +2\beta \right)\left[\left(\theta +\beta p_{c}e\right)\left(\theta +\beta p_{c}e+\omega +\gamma p_{c}e\right)-\left(\gamma +2\beta \right)h\right]}$$

$$\tau_{1}^{c}-\tau_{2}^{c}=\frac{\left(\lambda_{1}-\lambda_{2}\right)\left(\theta +\beta p_{c}e\right)\sigma }{\left(\theta +\beta p_{c}e\right)\left(\theta +\beta p_{c}e+\omega +\gamma p_{c}e\right)-\left(\gamma +2\beta \right)h}$$

when $h>\frac{\left(\theta +\beta p_{c}e\right)\left(\theta +\beta p_{c}e+\omega +\gamma p_{c}e\right)}{\gamma +2\beta }$, if $\lambda_{1}>\lambda_{2}$, then $p_{1}^{c}-p_{2}^{c}>0$, $\tau_{1}^{c}-\tau_{2}^{c}<0$; If $\lambda_{2}>\lambda_{1}$, then $p_{1}^{c}-p_{2}^{c}<0$, $\tau_{1}^{c}-\tau_{2}^{c}>0$.

When $h<\frac{\left(\theta +\beta p_{c}e\right)\left(\theta +\beta p_{c}e+\omega +\gamma p_{c}e\right)}{\gamma +2\beta }$, if $\lambda_{1}>\lambda_{2}$, then $p_{1}^{c}-p_{2}^{c}<0$, $\tau_{1}^{c}-\tau_{2}^{c}>0$; if $\lambda_{2}>\lambda_{1}$, then $p_{1}^{c}-p_{2}^{c}>0$, $\tau_{1}^{c}-\tau_{2}^{c}<0$. □

From Proposition 6, when the investment cost is high, there is a risk of technical failure, so manufacturers with higher risk aversion levels will buy carbon quotas to meet their carbon emission needs instead of increasing their carbon emission reduction technology investment. When the investment cost is low, manufacturers with higher risk aversion levels will increase their investment in technology rather than purchase carbon quotas. It is interesting that regardless of the cost factor of technology investment, the manufacturer with a higher carbon emission reduction rate will have a lower price, which is beneficial for both environmental protection and consumers.

### 5.2. Effect of Carbon Trading Price on Optimal Solutions

**Figure 3:**
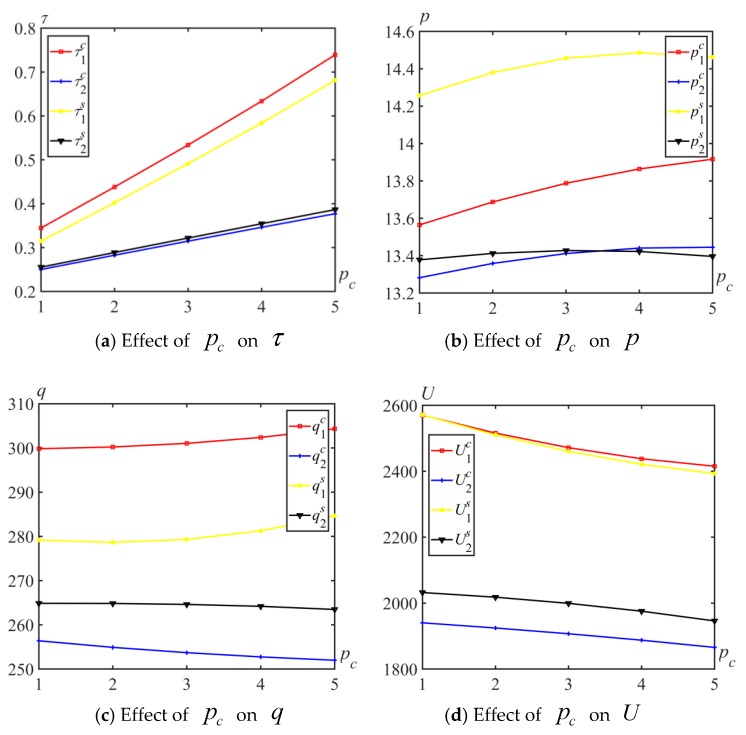
Effect of $p_{c}$ on $p_{i}$, $q_{i}$, and $U_{i}$.

Based on Table 2, $p_{c}$ was set between 1 and 5, and the effects of on the optimal emission reduction rate, price, expected quantity and utility for the two different competitive behaviors are shown in Figure 3. Several insights are obtained from Figure 3.

**Insight 3.**  *Regardless of the competitive models, increasing the carbon trading price* $p_{c}$ *raises the emission reduction level $\tau_{i}$* *and expected quantity* $q_{1}$ *, but decreases the expected* *quantity* $q_{2}$ *and utility* $U_{i}(i=1,2)$ *. When* $p_{c}$ *increases, the price* $p_{i}$ *increases to a certain level, and then, they will no longer keep rising. Therefore, while the carbon trading price increases, enterprises have a greater incentive to cut down emissions.*

**Insight 4.**  *For Manufacturer 1, the carbon emission reduction rate, expected quantity and utility for Cournot behavior are higher than those of Stackelberg behavior, but the production price is lower for Cournot behavior, that is,* $\tau_{1}^{c}>\tau_{1}^{s}$ *,* $p_{1}^{c}<p_{1}^{s}$ *,* $q_{1}^{c}>q_{1}^{s}$ *, and* $U_{1}^{c}>U_{1}^{s}$ *. For Manufacturer 2, when* $p_{c}$ *increases, the relation* $p_{2}^{c}<p_{2}^{s}$ *changes to* $p_{2}^{c}>p_{2}^{s}$ *, but the optimal emission reduction rate, expected quantity and utility for the Stackelberg behavior are higher than that of Cournot behavior, that is,* $\tau_{2}^{s}>\tau_{2}^{c}$ *,* $q_{2}^{s}>q_{2}^{c}$ *and* $U_{2}^{s}>U_{2}^{c}$ *. Therefore, Manufacturer 2 is willing to be a follower in Stackelberg behavior, but Manufacturer 1 prefers Cournot behavior.*

### 5.3. Effect of Carbon Quota on Optimal Solutions

**Figure 4:**
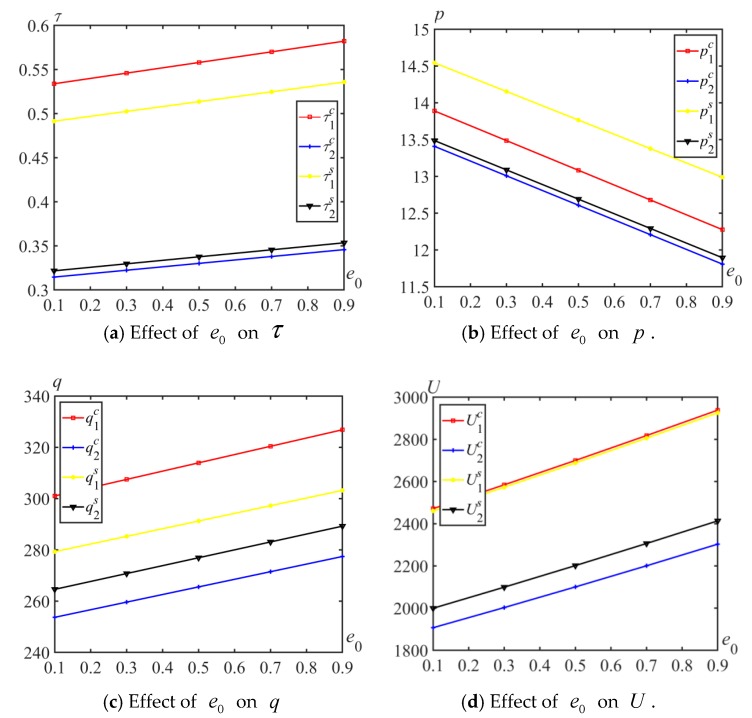
Effect of $e_{0}$ on $\tau_{i}$, $q_{i}$, and $U_{i}$.

When $e_{0}$ changes from 0.1 to 0.9, the effects of $e_{0}$ on the optimal price, emission reduction rate, expected quantity and utility for the two different competitive behaviors are shown in Figure 4, from which several insights are obtained in Figure 4.

**Insight 5.**  *No matter which competition model, if the carbon quota* $e_{0}$ *increases, the optimal carbon reduction rate* $\tau_{i}$ *, expected quantity* $q_{i}$ *and utility* $U_{i}$ *increase, but the optimal price* $p_{i}(i=1,2)$ *decreases. The increase in the carbon quota means that the government relaxes restrictions on carbon emissions, and manufacturers have no burden of carbon emission reduction. To attract more consumers, manufacturers adopt a strategy of lower prices. Because consumers are more concerned about the low carbon degree than the price of products, manufacturers are more willing to conduct carbon emission reduction to gain greater attention from consumers.*

**Insight 6.**  *When* $e_{0}$ *increases, for Manufacturer 1, the relations* $\tau_{1}^{c}>\tau_{1}^{s}$ *,* $p_{1}^{s}>p_{1}^{c}$ *,* $q_{1}^{c}>q_{1}^{s}$ *and* $U_{1}^{c}>U_{1}^{s}$ *are established. For Manufacturer 2, the relations* $\tau_{2}^{s}>\tau_{2}^{c}$ *,* $p_{2}^{s}>p_{2}^{c}$ *,* $q_{2}^{s}>q_{2}^{c}$ *and* $U_{2}^{s}>U_{2}^{c}$ *are established. The utility of Manufacturer 1 in Cournot behavior is very close to that in Stackelberg behavior. His/her utility is much greater than that of Manufacturer 2. Compared with Stackelberg behavior, Manufacturer 1 prefers Cournot behavior. However, Manufacturer 2 prefers Stackelberg behavior.*

### 5.4. Effect of Production Cost on Optimal Solutions

**Figure 5:**
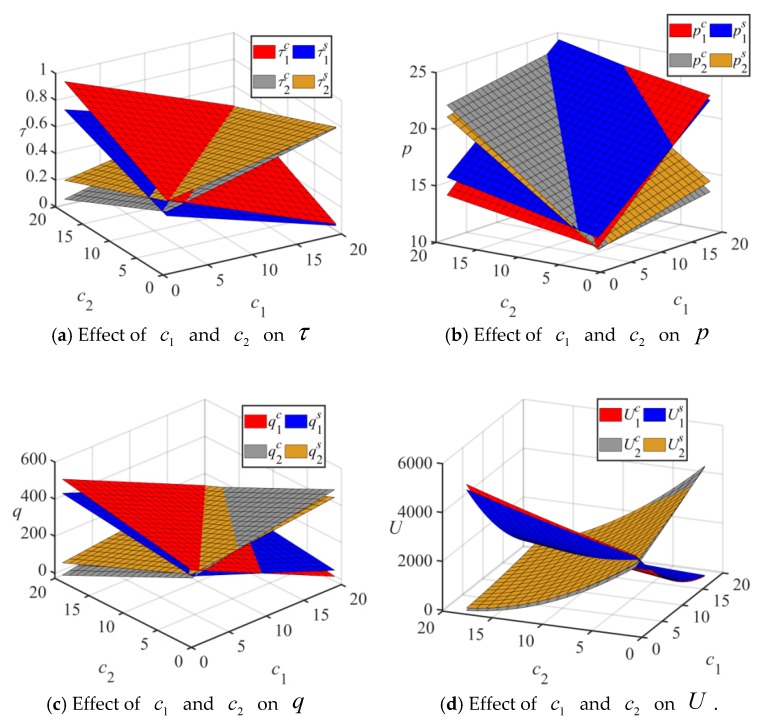
Effect of $c_{i}$ on $\tau_{i}$, $p_{i}$, $q_{i}$, and $U_{i}$.

Based on Table 2, $c_{i}$ is set between 1 and 20, and the effects of $c_{i}$ on the optimal price, carbon emission reduction rate, expected quantity and utility for the two different competitive behaviors are shown in Figure 5. Several insights can be obtained from Figure 5.

**Insight 7.**  *Regardless of the type of competition model, the manufacturer’s optimal carbon emission reduction rate, expected quantity and utility decrease in their own production cost and increase in their rival’s production cost. However, the price increases in production costs. Because capital is limited, with increasing production costs, manufacturers’ investment in technology decreases, which decreases the emission reduction rate. We can see that the lower production cost is beneficial to manufacturers for producing more low-carbon products and increases their utilities. Therefore, to produce more environmentally friendly products and obtain higher utilities, manufacturers need to find ways to reduce production costs.*

**Insight 8.**  *According to Assumption 1,*$c_{2}>c_{1}$. *Regardless of the type of competitive model, Manufacturer 1’s optimal emission reduction rate, expected quantity, and utility are all greater than those of Manufacturer 2, but the optimal price is lower. For Manufacturer 1,*τ*,*q*and*U*in the Cournot behavior model are always greater than those in the Stackelberg behavior model, but the price relation is the opposite, that is,*$\tau_{1}^{c}>\tau_{1}^{s}$*,*$p_{1}^{c}<p_{1}^{s}$*,*$q_{1}^{c}>q_{1}^{s}$*, and*$U_{1}^{c}>U_{1}^{s}$*. For Manufacturer 2, the relations*$\tau_{2}^{s}>\tau_{2}^{c}$*,*$p_{2}^{c}>p_{2}^{s}$*,*$q_{2}^{s}>q_{2}^{c}$*and*$U_{2}^{s}>U_{2}^{c}$*are established. Therefore, Manufacturer 2 prefers to be a follower in Stackelberg behavior, while Manufacturer 1 prefers Cournot behavior.*

## 6. Conclusions

In this paper, the optimal pricing and carbon emission reduction decisions of two competing manufacturers have been examined under the C&T policy. In the case of uncertain market demand, we assumed that manufacturers have different risk aversion levels. Two models under different competition behaviors were established. Through analysis and calculation, some conclusions were drawn.

From a theoretical perspective, we have obtained several conclusions. Firstly, regardless of whether in the Cournot behavior model or Stackelberg behavior model, the optimal decision and utility of the two manufacturers are chiefly influenced by the carbon quota, carbon trading price, risk aversion level and production cost. Secondly, when the carbon trading price increases, manufacturers’ optimal carbon emission reduction rate and price increase, but utilities decrease. Therefore, from an environmental perspective, the high carbon trading price will provide manufacturers an incentive to reduce carbon emissions, and from consumers’ perspective, they can buy low-carbon products at a low price. Third, when the carbon quota increases, manufacturers’ emission reduction rate and utilities increase, but the production price decreases. Fourth, taking into account the uncertainty of market demand, the risk aversion level of manufacturers has a significant impact. When manufacturers’ risk aversion levels decrease, their optimal carbon emission reduction rates and utilities all increase, but the price decreases. If they take a more optimistic attitude toward risk, they are more willing to reduce carbon emissions, which is beneficial for environmental protection, manufacturers and consumers. And fifth, the order of decision making also affects manufacturers’ optimal decisions. Manufacturer 1′s utility is almost equal for the two different competitive behaviors, but the carbon emission reduction rate is higher and the price is lower in the Cournot behavior model. For Manufacturer 2, compared with the Cournot behavior model, he/she makes more utility and higher carbon emission reduction rate in the Stackelberg behavior model as a follower. Therefore, small-scale manufacturers prefer Stackelberg behavior.

From a practical point of view, we also put forward some conclusions. Enterprises facing risks caused by uncertain market demand can obtain higher utilities when their risk aversion levels are low. It is necessary for manufacturers to use historical data and technical methods to predict product demand to reduce their risk aversion levels. Consumer environmental awareness increases the demand for low-carbon products, so it is better for manufacturers to have an optimistic attitude to actively reduce carbon emissions. The government has enacted policies to inspire enterprises to cut down carbon emissions. Although increasing the carbon quota can increase enterprises’ profits, as consumers’ environmental awareness increases, it is better for the government to reduce the carbon quota and to motivate manufacturers’ internal enthusiasm for emissions reduction. Therefore, the government ought to adopt effective policies that will be consistent with manufacturers’ advanced low-carbon technology and the consumers’ low-carbon preference.

The above conclusions provide suggestions for manufacturers and governments in terms of theory and practice. We also summarize some limitations of this study and propose possible research directions in the future. First, our research background is based on Assumption 1, which represents most of the current market conditions, such as traditional automobile enterprises and new energy automobile enterprises; it cannot summarize all of the actual scenarios in life and there will still be special situations. Therefore, our conclusion only applies to the scenario we have assumed, which is a limitation in practice. Second, the market demand is influenced by many complex factors in reality, so other forms of demand functions could be considered in future studies, non-linear form. Third, we set the carbon trading price and carbon quota as fixed parameters, which can be considered the decision-making variables of the government in future studies. Fourth, our paper focuses on manufacturer competition under the C&T scheme, and we can also consider further research in the context of other carbon emission policies, such as carbon taxes. In reality, upstream and downstream enterprises usually participate in carbon emission reduction activities; thus, a possible extension is to consider a two-echelon SC for carbon emission reduction problems.

## Figures and Tables

**Table 1:** Notations.

Symbol	Definitions
Parameters	
$d_{i}$/${\tilde{d}}_{i}$	Certain/uncertain initial market demand quantity of the product *i*
β	Self-price elasticity of demand
γ	Cross-price elasticity of demand
θ	Self-carbon emission elasticity
ω	Cross-carbon emission elasticity
Dependent Variables	
$q_{i}$/${\tilde{q}}_{i}$	Expected/uncertain market demand quantity of the product *i* or production quantity of the manufacturer *i*
$e_{0}$	Carbon quota per unit of product allocated by the government to the manufacturers
$e_{i}$	Initial unit carbon emissions of the manufacturer *i*
$c_{i}$	Unit production cost of manufacturer *i*
$p_{c}$	Unit price of carbon quota trading
$h_{}$	Cost coefficient of carbon emission reduction technology investment
$\lambda_{i}$	Risk aversion level of manufacturer *i*
Decision Variables	Definitions
$p_{i}$	Price of the product produced by manufacturer *i*
$\tau_{i}$	Carbon emission reduction rate per unit of production after carbon emission reduction technology investment

**Table 2:** Parameter values.

Parameters	$d_{1}$	$d_{2}$	β	γ	θ	ω	$e_{1}$	$e_{2}$	h	σ	$e_{0}$	$p_{c}$	$c_{1}$	$c_{2}$	$\lambda_{i}$
Value	500	450	30	15	40	25	0.5	0.2	1500	30	0.1	3	4	5	0.5
